# Coagulation Studies Are Not Predictive of Hematological Complications of COVID-19 Infection

**DOI:** 10.1055/s-0041-1742225

**Published:** 2022-01-17

**Authors:** Sarah Hadique, Varun Badami, Rahul Sangani, Michael Forte, Talia Alexander, Aarti Goswami, Adriana Garrison, Sijin Wen

**Affiliations:** 1 Department of Internal Medicine, Section of Pulmonary, Critical Care & Sleep Medicine, West Virginia University, Morgantown, West Virginia, United States; 2 Department of Epidemiology and Biostatistics, West Virginia University, Morgantown, West Virginia, United States; 3 Department of Pathology, Anatomy and Laboratory Medicine, West Virginia University, Morgantown, West Virginia, United States

**Keywords:** COVID-19, thrombosis, bleeding, ADAMTS-13, 30-day mortality

## Abstract

**Objectives** Thrombotic and bleeding complications are common in COVID-19 disease. In a prospective study, we performed a comprehensive panel of tests to predict the risk of bleeding and thrombosis in patients admitted with hypoxic respiratory failure due to severe COVID-19 infection.

**Methods** We performed a single center (step down and intensive care unit [ICU] at a quaternary care academic hospital) prospective study. Sequentially enrolled adult (≥18 years) patients were admitted with acute hypoxic respiratory failure due to COVID-19 between June 2020 and November 2020. Several laboratory markers of coagulopathy were tested after informed and written consent.

**Results** Thirty-three patients were enrolled. In addition to platelet counts, prothrombin time, and activated partial thromboplastin time, a series of protocol laboratories were collected within 24 hours of admission. These included Protein C, Protein S, Antithrombin III, ADAMTS13, fibrinogen, ferritin, haptoglobin, and peripheral Giemsa smear. Patients were then monitored for the development of hematological (thrombotic and bleeding) events and followed for 30 days after discharge. Twenty-four patients (73%) required ICU admissions. At least one laboratory abnormality was detected in 100% of study patients. Nine patients (27%) suffered from significant hematological events, and four patients had a clinically significant bleeding event requiring transfusion. No significant association was observed between abnormalities of coagulation parameters and the incidence of hematologic events. However, a higher SOFA score (10.89 ± 3.48 vs. 6.92 ± 4.10, *p* = 0.016) and CKD (5/9 [22.2%] vs. 2/24 [12.5%] *p* = 0.009) at baseline were associated with the development of hematologic events. 33.3% of patients died at 30 days. Mortality was similar in those with and without hematological events. Reduced ADAMTS13 level was significantly associated with mortality.

**Conclusion** Routine extensive testing of coagulation parameters did not predict the risk of bleeding and thrombosis in COVID-19 patients. Thrombotic and bleeding events in COVID-19 patients are not associated with a higher risk of mortality. Interestingly, renal dysfunction and a high SOFA score were found to be associated with increased risk of hematological events.

## Introduction

Since its initial declaration as a pandemic in March 2020, COVID-19 syndrome has been associated with a wide array of clinical consequences. 1 In addition to the human toll, one of the major concerns is the direct medical cost and resource use burden imposed on the health care system. 2 3 Morbidity and mortality from COVID-19 infection is not only restricted to acute respiratory failure but also include cardiovascular compromise, neurological dysfunction, multiorgan failure, life-threatening sepsis, and COVID-19-associated coagulopathy with serious thrombotic complications. 4 5 Hematologic abnormalities in severe COVID-19 disease have gained interest, as the implications of coagulopathy and thrombotic disorders are wide ranging and evidence continues to mount in both clinical reports and autopsy studies. Pulmonary embolism (PE) and deep vein thrombosis (DVT) are the most often noted thrombotic events in patients with COVID-19 with incidence rate reported to be 23% in a recent meta-analysis. 6 Moreover, autopsy studies have noted macrovascular and microvascular fibrinous thrombi in the lungs and glomerular capillary bed, some of which were consistent with compliment-mediated microvascular injuries. 7 8 9 10 Arterial events have also been noted to occur at increased rates, with a recent study from New York reporting a 1.6% stroke rate and 8.9% myocardial infarction rate. 11

Though the complete pathophysiologic mechanism behind COVID-related hypercoagulability has not yet been entirely elucidated, the components of Virchow's triad (endothelial injury, stasis, and hypercoagulable state) are present in some form in severe COVID disease. Iba et al have recently reviewed our current understanding of COVID-19-associated coagulopathy. 12 However, more work is needed to fully understand the incidence and clinical significance of coagulopathy among COVID-19 patients with acute respiratory failure. Autopsy studies have shown endothelial inflammation occurring in the pulmonary, renal, hepatic, and cardiac vasculature. 13 14 15 In fact, evidence suggests that the cytokine storm, which is itself associated with severe COVID-19 disease and implicated in rapid progression of disease, may be caused or worsened by endothelial inflammation and exocytosis. 16 Immobilization from severe hypoxic respiratory failure and prolonged hospital stay often complicates the care of COVID-19 patients. 17 The final part of the triad, hypercoagulability, is also under investigation. Wide ranging reports of abnormalities in routine coagulation testing, 18 viscosity, 19 and even thromboelastography 20 have supported the presence of multiple levels of coagulation abnormalities. There is a need to understand the rate of bleeding and thrombotic manifestations associated with COVID-19 coagulopathy, as well as the clinical utility of abnormal coagulation testing to predict risk for bleeding, thrombosis, and severity of illness. Frequent laboratory testing contributes to overall cost of care. Given the magnitude of disease burden, every effort must be made to minimize the direct cost of care of patients admitted with COVID-19 infection.

A single center, prospective investigation was conducted to measure both conventional and specific procoagulant tests in hospitalized patients with severe COVID-19 infections, to assess how often abnormalities in coagulation parameters occur in these patients and whether any of these abnormalities are associated with the future development of clinically significant hematologic events.

## Materials and Methods

### Design and Protocol

This is a prospective single center study conducted in an academic quaternary medical center of patients admitted with severe COVID-19 infection and acute hypoxemic respiratory failure. Institutional review board approval was obtained (IRB # 2005006386). Research reported in this publication was supported by The National Institute of General Medical Sciences of The National Institutes of Health under Award Number 5U54GM104942–05. Electronic medical record was reviewed to identify consecutive adult hypoxic patients admitted within the first 24 hours with a confirmed COVID-19 reverse transcriptase-polymerase chain reaction (RT-PCR), after which the investigators obtained informed consent from the patient or their surrogate (in person or via telephone). Then a panel of laboratory studies was performed, henceforth called “protocol laboratories” within 24 hours of admission. Only one set of protocol laboratories was collected per patient. The treating physicians were not blinded to the results of these laboratories. Once the patients were enrolled, they were followed during the hospitalization and 30 days after discharge. Any hematological event, which happened during the hospitalization, was recorded.

The primary outcome of the study was to assess whether abnormalities in the “protocol laboratories” were able to predict COVID-19 related hematologic events, and to describe the rate of bleeding and thrombotic complications in a cohort of critically and non-critically ill hospitalized COVID-19 patients. We defined hematologic events as venous or arterial thrombosis—DVT, PE, arterial clot, cerebrovascular accident, and acute coronary syndrome, filter clotting of renal replacement therapy, and bleeding requiring transfusion. Moderate or severe bleeding events as per the Gusto definition were included in the study. 21 All the patients with bleeding events had concomitant thrombosis as well. Synchronously diagnosed DVT and PE or bleeding complication requiring blood transfusion in the same patient were considered as one hematologic event. PE, DVT, and arterial clots were confirmed radiographically. We divided patients into two groups—patients with hematologic events were compared with patients without such events.

The secondary outcome was to compare mean intensive care unit (ICU) length of stay (LOS), hospital LOS, severity of illness, and 30-day mortality between the groups with hematological events and with those no events. Severity of illness was defined as Sequential Organ Failure Assessment (SOFA) score, P/F ratio, use of high flow nasal cannula (HFNC), use of non-invasive positive pressure ventilation (NIPPV), use of invasive mechanical ventilation (IMV), or requirement of renal replacement therapy.

### Laboratory Investigations

The “protocol laboratories” drawn from consented patients were protein C, protein S, antithrombin III level, A disintegrin and metalloproteinase with a thrombospondin type 1 motif, member 13 (ADAMTS13), haptoglobin, and peripheral (Giemsa) smear. Other collected data included prothrombin time (PT), activated partial thromboplastin time (aPTT), international normalized ratio (INR), D-dimer, fibrinogen, ferritin, lactate dehydrogenase (LDH), complete blood counts (CBCs), basic metabolic panel, and hepatic function testing. ADAMTS13 and plasma protein S activity were performed at the Mayo Clinic Reference laboratory, which developed and determined the reference ranges as follows: protein S activity normal range 65 to 160%, ADAMTS13 range ≥70%. Plasma protein C activity, antithrombin III functional profile, and haptoglobin were performed at the West Virginia University Clinical Laboratory, with reference ranges as follows: Protein C activity range 70 to 140%, antithrombin III functional profile reference 83 to 128%, and haptoglobin >32 mg/dL. Peripheral smears were reviewed by the pathologist.

### Population and Data Collection

Inclusion criteria were hospitalized adult patients (≥18 years old) with hypoxic respiratory failure and positive COVID-19 RT-PCR within 24 hours of the admission. Hypoxic respiratory failure was defined as arterial saturation ≤88% requiring supplemental oxygen. Exclusion criteria were age <18 years, pregnancy, incarceration, incidental finding of COVID-19 without concomitant hypoxia, or inability to obtain informed consent from the patient or health care surrogate within the first 24 hours of admission.

Baseline demographics, comorbid conditions, measures of illness, and hypoxia were collected. The measures of illness included Charlson Comorbidity Index (CCI), SOFA scores, requirement of HFNC, NIPPV, requirement and length of mechanical ventilation, and P/F or S/F ratios. Data was aggregated using the HIPAA-compliant Research Electronic Data Capture (REDCap) electronic data capture tool. 22 23 Thirty-four patients were enrolled in the study; however, one patient was excluded due to incomplete protocol laboratories. Thirty-three patients were divided into two groups based on the presence or absence of hematologic events.

### Statistical Analysis

All data analyses were performed using the statistical software R, version 3.6.3. Mean and standard deviations were reported for continuous variables, and proportions for categorical variables. Differences between groups were assessed by the Wilcoxon rank test for continuous variables and Fisher exact test for categorical variables. Protocol laboratories were analyzed as continuous variables and also by stratifying them as dichotomous (normal vs. abnormal). Kaplan-Meier method and log-rank test were used to assess 30-day mortality difference between the two groups. All statistical tests were two-sided, and an *α* -value of 0.05 was used to determine statistical significance.

## Results

### General Characteristics

Thirty-three consecutive patients were recruited for this prospective study evaluating hematological impact of severe COVID-19 infection requiring hospital admission between June 2020 and November 2020. Twenty-four patients (73%) required intensive care unit admissions. Nine patients (27%) suffered from significant hematological events. In contrast, four patients had a clinically significant bleeding event requiring transfusion. All patients in the study were on venous prophylaxis.

The mean age was 68.48 ± 13.89 and mean BMI 32.56 ± 5.98. 63.6% of patients were male. There was no difference of age, gender, or BMI between patients with or without hematologic events ( Table 1 ). Prevalent comorbidities included hypertension (75.7%), hyperlipidemia (72.7%), diabetes (57.6%), coronary artery disease (CAD, 39.4%), and COPD (15.2%), with no significant difference between the groups. Chronic kidney disease (CKD) was more common in the group with hematological events (55.6 vs. 8.3%, *p* = 0.009). Use of prophylaxis and full dose anticoagulation with either subcutaneous unfractionated heparin or low molecular weight heparin was similar between the groups.

**Table 1:** Characteristics of groups with and without combined thrombosis and bleeding events

Variables Mean ± SD or *n* (%)	Group with thrombosis and/or bleeding events ( *n* = 9)	Group without thrombosis and/or bleeding events ( *n* = 24)	Total cohort ( *n* = 33)	*p* -Value
Age	70.2 ± 10.7	67.8 ± 15.1	68.5 ± 13.9	0.761
Gender, Male *n* (%)	6 (66.7%)	15 (62.5%)	21 (63.6%)	1
BMI	34.3 ± 4.3	31.9 ± 6.5	32.6 ± 6.0	0.29
CCI	7 ± 3.5	5 ± 2.3	5.6 ± 2.8	0.053
Comorbidities, %				
Hypertension	7 (77.8%)	18 (75%)	25 (75.8%)	1
Hyperlipidemia	6 (66.7%)	18 (75%)	24 (72.7%)	0.677
Diabetes type II	6 (66.7%)	13 (54.2%)	19 (57.6%)	0.698
CAD	6 (66.7%)	7 (29.2%)	13 (39.4%)	0.107
CHF	0 (0%)	2 (8.3%)	2 (6.1%)	1
CKD	5 (55.6%)	2 (8.3%)	7 (21.2%)	***0.009***
COPD	2 (22.2%)	3 (12.5%)	5 (15.2%)	0.597
Home use of anticoagulation	1 (11.1%)	2 (8.3%)	3 (9.1%)	1
Severity of illness				
SOFA	10.9 ± 3.5	6.9 ± 4.1	8.0 ± 4.3	***0.016***
P/F ratio	95.4 ± 44.3	140.3 ± 82.7	128.0 ± 76.3	0.189
Use of HFNC, %	5 (55.5%)	15 (62.5%)	20 (60.6%)	1
Use of NIV-PPV, %	6 (66.7%)	15 (62.5%)	21 (63.6%)	1
Invasive mechanical ventilation	6 (66.7%)	9 (37.5%)	15 (45.5%)	0.239
Hemodialysis	3 (33.3%)	1 (4.2%)	4 (12.1%)	0.052
Inpatient medications, %				
Subcutaneous heparin	5 (55.6%)	4 (16.7%)	9 (27.3%)	0.073
Subcutaneous LMWH	3 (33.3%)	17 (70.8%)	20 (60.6%)	0.107
Full dose anticoagulation	4 (44.4%)	10 (41.7%)	14 (42.4%)	1
a, Unfractionated heparin	3 (33.3%)	4 (16.7%)	7 (21.2%)	0.358
b, LMWH	1 (11.1%)	6 (25%)	7 (21.2%)	0.642
Warfarin	1 (11.1%)	1 (4.2%)	2 (6.1%)	0.477
Aspirin	8 (88.9%)	9 (37.5%)	17 (51.5%)	***0.017***
Other antiplatelet agents	4 (44.4%)	2 (8.3%)	6 (18.2%)	***0.034***
Outcomes				
ICU length of stay	11.8 ± 15.5	7.6 ± 9.4	8.8 ± 11.3	0.297
Hospital length of stay	20.7 ± 16.5	12.9 ± 10.2	15.0 ± 12.4	0.120
30-day mortality	3 (33.3%)	8 (33.3%)	11 (33.3%)	1

Abbreviations: BMI, body mass index; CAD, coronary artery disease; CCI, Charlson comorbidity index; CHF, congestive heart failure; CKD, chronic kidney disease; COPD, chronic obstructive pulmonary disease; HFNC, high flow nasal cannula; LMVH, low molecular weight heparin; NIV-PPV, noninvasive positive pressure ventilation; P/F ratio, arterial pO2 divided by the fraction of inspired oxygen; SOFA, sequential organ failure assessment.

### Disease Severity

A higher SOFA score was observed in the group with hematological events (10.89 ± 3.48 vs. 6.92 ± 4.10, *p* = 0.016). Though the group with hematological events was noted to have a lower PaO _2_ /FiO _2_ (P/F) ratio, it did not reach statistical significance (95.44 ± 44.27 vs. 140.25 ± 82.67, *p* = 0.189). Use of HFNC, non-invasive ventilation (NIV), and IMV was not different between the groups. Approximately half of the patients (45.5%) required IMV. A subset of patients (4/33, 12.2%) required hemodialysis, with a trend toward higher need in the hematological events group (3/9 vs. 1/24, *p* = 0.052). See Table 1 for characteristics of the total cohort and groups of hematologic events versus no events.

### Laboratory Results

Groups with hematological events showed significantly lower hemoglobin (10.8 ± 1.58 g/dL vs. 12.6 ± 1.9 g/dL, *p* = 0.019) and fibrinogen (552.5 ± 147.2 mg/dL vs. 706.9 ± 145.8 mg/dL, *p* = 0.041), respectively. Moreover, the group with hematologic events had evidence of worsened renal function, with significantly higher blood urea nitrogen (51.3 ± 26.8 mg/dL vs. 23.9 ± 21.3 mg/dL, *p* = 0.005) and creatinine (3.3 ± 2.1 mg/dL vs. 1.1 ± 0.6, *p* < 0.001). Mean LDH values trended higher in the group with hematological events. Our entire cohort exhibited marked abnormalities with elevated values of ferritin (87.7%), D-dimer (72.6%), PT/INR (31.2%), and lower values of fibrinogen (69.6%), without significant differences between groups. Interestingly, one-fifth of the patients were noted to have reduced protein C activity, antithrombin III function, and ADAMTS-13 concentrations. At least one abnormality in coagulation parameter was found in 100% of study population. Table 2 provides details of laboratory values as continuous variables whereas Table 3 describes the frequencies of abnormal protocol laboratories between the groups.

**Table 2:** Laboratory variables of groups with and without combined thrombosis and bleeding events

Laboratory variables (units) Mean ± SD	Group with thrombosis and/or bleeding events ( *n* = 9)	Group without thrombosis and/or bleeding events ( *n* = 24)	*p* -Value
WBC (x10 ^3^ /uL)	10.6 ± 4.8	9.7 ± 5.3	0.44
Hemoglobin (g/dL)	**10.8 ± 1.6**	**12.6 ± 1.9**	***0.019***
Hematocrit (%)	33.5 ± 5.0	37.5 ± 4.5	0.055
Platelets (x10 ^3^ /uL)	258.5 ± 109.2	202.8 ± 67.8	0.207
BUN (mg/dL)	**51.33 ± 26.83**	**23.87 ± 21.27**	***0.005***
Creatinine (mg/dL)	**3.40 ± 2.06**	**1.08 ± 0.56**	***<0.001***
Total protein (g/dL)	6.58 ± 0.79	6.38 ± 1.01	0.714
Albumin (g/dL)	2.53 ± 0.54	2.75 ± 0.47	0.239
Total bilirubin (mg/dL)	0.44 ± 0.19	0.63 ± 0.35	0.144
Direct bilirubin (mg/dL)	0.28 ± 0.12	0.31 ± 0.18	0.929
AST (U/L)	78.56 ± 65.88	59.16 ± 83.26	0.082
ALT (U/L)	43.0 ± 39.6	54.83 ± 78.20	0.479
Alkaline phosphatase (U/L)	120.44 ± 81.67	79.37 ± 41.86	*0.041*
LDH (U/L)	646.25 ± 202.89	485.36 ± 150.46	0.057
PT (seconds)	21.76 ± 22.45	13.97 ± 3.52	0.179
INR	1.98 ± 2.26	1.21 ± 0.28	0.215
aPTT (seconds)	42.32 ± 32.20	30.042 ± 7.05	0.605
Fibrinogen (mg/dL)	**552.5 ± 147.2**	**706.88 ± 145.84**	***0.041***
D-dimer (ng/mL DDU)	735.75 ± 386.20	1004.18 ± 598.52	0.344
Ferritin (ng/mL)	1089.37 ± 409.49	1033.69 ± 1066.25	0.172
Haptoglobin (mg/dL)	428.22 ± 141.30	390.61 ± 100.21	0.415
Protein C (IU/dL)	87.55 ± 38.24	94.25 ± 21.52	0.642
Protein S (%)	95.0 ± 38.58	103.0 ± 24.46	0.652
Antithrombin III (%)	101.55 ± 11.39	93.5 ± 17.88	0.203
ADAMTS13 (%)	91.22 ± 13.35	85.58 ± 18.15	0.506

Abbreviations: ADAMST13, A Disintegrin and Metalloproteinase with a Thrombospondin type 1 motif, member 13, also known as von Willebrand factor-cleaving protease (VWFCP); ALT, alanine transaminase; aPTT, activated partial thromboplastin time; AST, aspartate transaminase; BUN, blood urea nitrogen; INR, international normalized ratio; LDH, lactate dehydrogenase; PT, prothrombin time; WBC, white blood cell.

**Table 3:** Abnormal protocol laboratories between the groups with and without thrombosis and/or bleeding events

Protocol laboratories (abnormal values), *n* (%)	Group with thrombosis and/or bleeding events *n* = 9 (%)	Group without thrombosis and/or bleeding events *n* = 24 (%)	Total cohort *N* = 33 (%)	*p* -Value
Elevated PT/INR	4 (44.4%)	6 (25%)	10 (30.3%)	0.407
Elevated aPTT	2 (22.2%)	1 (4.2%)	3 (9.1%)	0.234
Reduced fibrinogen	6 (66.7%)	17 (70.8%)	23 (69.7%)	0.093
Elevated D-dimer	6 (66.7%)	18 (75%)	24 (72.7%)	0.645
Reduced protein C	4 (44.4%)	3 (12.5%)	7 (21.2%)	0.068
Reduced protein S	1 (11.1%)	1 (4.2%)	2 (6.1%)	0.456
Reduced antithrombin III	1 (11.1%)	7 (29.2%)	8 (24.2%)	0.394
Reduced ADAMST13	1 (11.1%)	6 (25%)	7 (21.2%)	0.642
Elevated ferritin	8 (88.9%)	21 (87.5%)	29 (87.9%)	1

* Abnormal values defined as: Reduced Platelets <150 × 10 ^3^ /uL; Elevated prothrombin time (PT) > 13.9 seconds; Elevated activated partial thromboplastin time (aPTT) >37.5 seconds; Reduced fibrinogen level <100 mg/dL; Reduced protein C level <70%; Reduced protein S level <65%; Reduced antithrombin III level <83%; Reduced ADAMST13 activity assay <70%; Elevated D-dimer (ng/mL DDU) >2 × upper limit of normal.

### Peripheral Smear Results

Of the 33 cases, CBC abnormalities included leukocytosis (39%) with absolute neutrophilia (52%), lymphopenia (67%), and monocytopenia (10%). In addition to normocytic anemia (58%), a single case displayed macrocytic anemia. The platelet counts varied greatly from thrombocytopenia (12%) to normal count (76%) to thrombocytosis (12%).

Thirty cases had peripheral smears available for review. Mild toxic changes with features such as vacuolization and granulation in the neutrophils (83%) were detected. Left shift to include meta-myelocytes or myelocytes was present in 13% of cases. Reactive lymphocytes were seen with changes, including moderate light blue cytoplasm (17%) and some cases with large granules (13%). The platelet morphologies showed anomalies, including large platelet forms (20%), platelet clumps (17%), or both (3%). Furthermore, anisopoikilocytosis was present in 10% of cases, notable for echinocytes; however, no schistocytes were seen. The leukoerythroblastic reaction was seen in one case. Monocytes, eosinophils, and basophils had normal morphologies in all cases.

### Primary Outcome

There was no statistically significant association between abnormal protocol laboratory values and hematologic events. Nine out of 33 (27%) patients suffered from clinically significant hematological events.

### Secondary Outcome

**Fig. 1:**
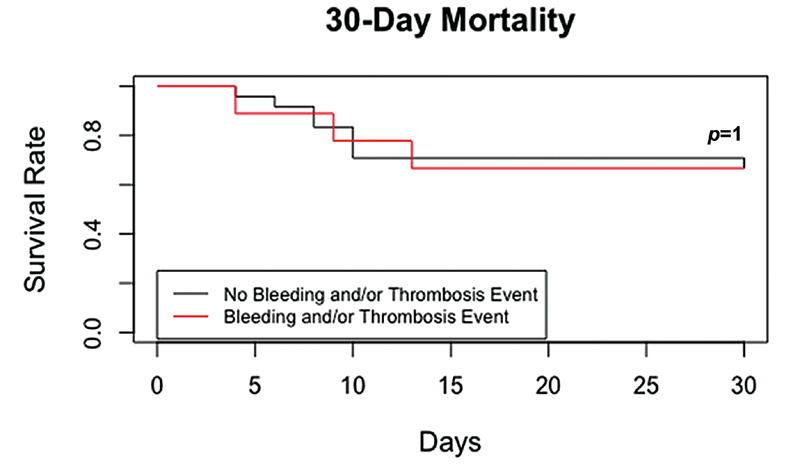
Kaplan-Meier curve of 30-day mortality. Net mortality was 33%.

Measured outcomes of mean ICU LOS, hospital LOS, and 30-day mortality were not different between the groups. Mean ICU and hospital LOS was 8.75 ± 11.31 and 15.03 ± 12.44, respectively in our cohort. 33.3% of patients were deceased at 30 days. Univariate analysis demonstrated the following significant prognostic predictors for 30-day mortality ( Table 4 ): SOFA score, use of IMV, days on mechanical ventilation, P/F ratio, WBC count, and ADAMTS-13 deficiency. Log-rank test did not show a statistically significant difference in 30-day mortality between the two groups ( *p* -value = 1). Fig. 1 shows the Kaplan-Meier curves of the groups of patients with and without hematologic abnormalities regarding 30-day mortality.

**Table 4:** Univariate analysis of significant prognostic factors associated with 30-day mortality in the total cohort ( *n* = 33)

Variables Mean ± SD or *n* (%)	Alive ( *n* = 22)	Dead ( *n* = 11)	*p* -Value
SOFA	6.1 ± 3.7	11.7 ± 2.6	*<0.001*
Invasive mechanical ventilation	6 (27.3%)	9 (81.8%)	*0.008*
Days on mechanical ventilation	2.9 ± 6.1	7.7 ± 5.6	*0.007*
P/F ratio	154.2 ± 80.7	75.7 ± 20.9	*0.003*
WBC count	8.46 ± 4.44	12.94 ± 5.27	*0.013*
ADAMTS-13 deficiency	2 (9.1%)	5 (45.5%)	*0.027*

Abbreviations: SOFA, Sequential Organ failure Assessment; WBC, white blood cell.

## Discussion

The high health care costs for COVID-19 patients represent a significant public health challenge. 24 Effective COVID-19 treatments for hospitalized patients may not only reduce disease burden but also ensure cost-effective practice and incorporation of the most relevant clinical parameters. 25 In our study, we found 100% of patients had at least one abnormality on laboratory parameter used for assessing coagulopathy. In addition, significant thrombotic and/or bleeding events were prevalent (27%) in hospitalized hypoxic COVID-19 patients, which is in accordance with larger retrospective studies of patients with COVID-19 disease. 6 Although thrombogenicity of COVID-19 differs considerably from other severe infectious and non-infectious diseases, 12 increased bleeding risk, especially in severely ill patients, remains a serious concern because bleeding complications are facilitated by thrombocytopenia, platelet dysfunction, and coagulation factor deficiencies, 26 27 which are often present in critically ill patients with COVID-19.

We found baseline renal dysfunction and elevated BUN and creatinine values were associated with increased hematologic events. Prior studies have shown that CKD and acute renal dysfunction confer approximately one-and-a-half-fold increase in incidence of venous thrombosis. The exact mechanism in which renal failure is a risk factor for hematological abnormalities is unknown but has been postulated to stem from endothelial dysfunction, platelet function defect, and enhanced activation and utilization of the coagulation system. 28 29 Increased severity of illness as defined by SOFA score was also seen in study to be independently associated with increased hematological events. A recent study revealed that the higher SOFA along with elevated D Dimer and hypoalbuminemia has been seen as independent risk factors of DVT. 30 Finally, demographics, CCI, and other markers of severity of illness (P/F ratio, use of HFNC, NIV PPV, IMV, and hemodialysis) were not significantly associated with hematological events.

Our study did not find any correlation between the abnormal protocol laboratory values and hematological events of thrombosis and/or clinically significant bleeding. An elevated D-dimer concentration is widely accepted as a specific coagulation abnormality in patients with COVID-19. A rising D-dimer concentration suggests a hypercoagulable state and microthrombus formation, and increased rates of VTE have been reported in ICU patients with COVID-19. 31 Of note, while most patients in this study had elevated levels of D-dimer, the level of elevation did not predict incidence of thrombosis. Lower fibrinogen levels were seen in patients with hematological events (552.5 ± 147.2 vs. 706.88 ± 185.84); though they were still above the upper limit of normal. There was no evidence that DIC (International Society on Thrombosis and Hemostasis criteria) was related to incidence of hematologic events, as we did not demonstrate a consumption of coagulation factor in our cohort or the presence of schistocytes on peripheral smear.

Our findings on peripheral smear included leukocytosis with absolute neutrophilia and absolute lymphopenia, in addition to monocytopenia. Toxic changes were seen in the neutrophils and larger granules were found in lymphocytes, similar to the findings described in Singh et al. 32 Furthermore, the presence of immature forms of granulocytes, meta-myelocytes, and myelocytes on peripheral smears has also been documented in literature. 33 34 35 Notably, detailed analysis of peripheral smears failed to identify the schistocytes. This finding is contrary to a recent study describing significant numbers of schistocytes in its cohort (17.6% overall) which was associated with increased mortality. Sweeney et al suggested that these findings, as well as significantly elevated levels of other hemolysis products, suggest evidence for a secondary thrombotic microangiopathy in COVID-19. 36 Our study does not confirm these findings.

Univariate analysis showed that patients with worse measures of disease severity including SOFA, P/F ratio, or requirement of IMV support, and days on MV had significantly worse mortality, which are known prognostic factors for worse outcomes in ARDS. 37 38 Finally a low ADAMTS13 level was associated with higher mortality in our study. Previous investigations have implicated ADAMTS13 levels as a marker for elevated risk of thrombosis and mortality. In 2007, two studies showed that serum levels of ADAMTS13 were lower in severe sepsis and septic shock, but had mixed evidence of mortality effects. 39 40 More recently, multiple studies demonstrated that patients with COVID-19 pneumonia have reduced levels of ADAMTS13, causing a microangiopathic thrombotic state 41 42 and leading to associations with worsened mortality. 36 43 44 Our study corroborates that ADAMTS13 deficiency was associated with mortality, but it failed to identify those patients with increased risk of hematologic events. Further, despite a low ADAMST13 levels in 21% of our patients, thrombotic thrombocytopenic purpura was not diagnosed in any patient in our study.

The strength of the study was its prospective design that allowed us to collect all basic and protocol laboratories within the first 24 hours of admission before any thrombotic and bleeding events. The analysis of protocol laboratories were conducted in-depth as both continuous and categorical variables (normal vs. abnormal). Independent pathologist reviewed peripheral smears. Limitations of the study included single center study with small sample size, non-blinded nature, lack of longer outpatient follow-up, and lack of ethnic diversity. The results should be regarded with some degree of caution due to lack of thromboembolism screening for all patients. If VTE screening had been applied, the incidence may have been even higher. Lastly, unmeasured confounding factors may have contributed to the findings of the study.

## Conclusion

Routine testing for protein C, protein S, anti-thrombin III, ADAMST13, and other routine coagulation parameters did not predict hematological events or adverse patient outcomes. Unless there is a strong suspicion for TTP, there is no therapeutic implication of isolated finding of a low ADAMST13 level. We suggest that routine ordering of special tests to assess the risk of thrombosis and bleeding in COVID-19 patients may not be indicated. Finding of high SOFA score and renal dysfunction with increased risk of hematological event needs to be confirmed on large sample size.
